# The Frequency and Energy of Snoring Sounds Are Associated with Common Carotid Artery Intima-Media Thickness in Obstructive Sleep Apnea Patients

**DOI:** 10.1038/srep30559

**Published:** 2016-07-29

**Authors:** Guo-She Lee, Li-Ang Lee, Chao-Yung Wang, Ning-Hung Chen, Tuan-Jen Fang, Chung-Guei Huang, Wen-Nuan Cheng, Hsueh-Yu Li

**Affiliations:** 1 Faculty of Medicine, School of Medicine, National Yang-Ming University, Taipei 11221, Taiwan, ROC; 2 Department of Otolaryngology, Taipei City Hospital, Ren-Ai Branch, Taipei 10629, Taiwan, ROC; 3 Department of Otorhinolaryngology - Head and Neck Surgery, Sleep Center, Linkou-Chang Gung Memorial Hospital, Tao-Yuan 33305, Taiwan, ROC; 4 Faculty of Medicine, College of Medicine, Chang Gung University, Taoyuan 33303, Taiwan, ROC; 5 Department of Cardiology, Sleep Center, Linkou-Chang Gung Memorial Hospital, Tao-Yuan 33305, Taiwan, ROC; 6 Department of Thoracic Medicine, Sleep Center, Linkou-Chang Gung Memorial Hospital, Tao-Yuan 33305, Taiwan, ROC; 7 Department of Laboratory Medicine, Linkou-Chang Gung Memorial Hospital, Tao-Yuan 33305, Taiwan, ROC; 8 Department of Medical Biotechnology and Laboratory Science, College of Medicine, Chang Gung University, Tao-Yuan 33303, Taiwan, ROC; 9 Graduate Institute of Biomedical Sciences, College of Medicine, Chang Gung University, Tao-Yuan 33303, Taiwan, ROC; 10 Department of Sports Sciences, University of Taipei, Tai-Pei 11153, Taiwan, ROC; 11 Department of Sleep Medicine, Royal Infirmary of Edinburgh, Edinburgh EH16 4SA, UK

## Abstract

Obstructive sleep apnea (OSA) is a known risk factor for atherosclerosis. We investigated the association of common carotid artery intima-media thickness (CCA-IMT) with snoring sounds in OSA patients. A total of 30 newly diagnosed OSA patients with no history of cardiovascular diseases were prospectively enrolled for measuring mean CCA-IMT with B-mode ultrasonography, body mass index, metabolic syndrome, 10-year cardiovascular disease risk score, high-sensitivity C-reactive protein, and homocysteine. Good-quality signals of full-night snoring sounds in an ordinary sleep condition obtained from 15 participants were further acoustically analyzed (Included group). All variables of interest were not significantly different (all *p* > 0.05) between the included and non-included groups except for diastolic blood pressure (*p* = 0.037). In the included group, CCA-IMT was significantly correlated with snoring sound energies of 0–20 Hz (*r* = 0.608, *p* = 0.036) and 652–1500 Hz (*r* = 0.632, *p* = 0.027) and was not significantly associated with that of 20–652 Hz (*r* = 0.366, *p* = 0.242) after adjustment for age and sex. Our findings suggest that underlying snoring sounds may cause carotid wall thickening and support the large-scale evaluation of snoring sound characters as markers of surveillance and for risk stratification at diagnosis.

A significantly increased risk of cardiovascular and all-cause mortality has been documented in subjects with obstructive sleep apnea (OSA)^1^,^2^,^3^,^4^. In addition, coexisting metabolic syndrome (MetS) and obesity in patients with OSA can have adverse cardiovascular consequences, including endothelial and vascular dysfunction^5^,^6^,^7^. Intermittent hypoxemia/reoxygenation during sleep may activate various pathophysiological mechanisms that may result in or exacerbate cardiovascular diseases (CVDs)^8^,^9^. These mediators − including inflammation, oxidative stress, sympathetic activation, hypercoagulability, and metabolic dysregulation − have proven roles in the pathogenesis of endothelial dysfunction^10^,^11^,^12^,^13^,^14^,^15^.

Recently, the association of carotid artery atherosclerosis with snoring and OSA has been the subject of intense research^16^,^17^,^18^,^19^,^20^,^21^. In animal models, the vibratory energy of snoring sounds can be transmitted to the carotid artery inducing endothelial dysfunction and atherosclerosis^22^,^23^,^24^ but does not directly alter baroreflex sensitivity^25^. In human studies, heavy snoring (>50% night snoring) may act as an independent risk factor for carotid atherosclerosis^16^. Notably, self-reported snoring is significantly associated with increased intima-media thickness (IMT) of the common carotid artery (CCA)^17^,^18^ but is not significantly related to carotid plaques^17^. Moreover, in cross-sectional studies, CCA-IMT was higher in OSA patients with an increased snoring index or MetS compared with patients with primary snoring^19^,^20^. However, during a four-year follow-up, the subclinical progression of carotid atherosclerosis did not seem to be accelerated by snoring^21^. Accordingly, snoring may participate in the early stages of carotid atherosclerosis (increased CCA-IMT) and slowly exacerbate this condition.

Previous studies assessed snoring using questionnaires^17^,^18^,^21^, snoring indexes^16^,^19^,^20^, and ratios of night snoring^16^ but have been criticized by some professionals for lacking a thorough analysis of other sound characteristics (such as frequency and energy)^26^. In this scenario, the relationship between snoring sound and atherosclerosis in OSA remains to be investigated. We tested the hypothesis that increased snoring sound energy of specific frequency domains could be associated with increased CCA-IMT in OSA patients. The possible mechanisms of snoring-associated atherosclerosis are also discussed.

## Results

### Study population

Of the 37 eligible adults, seven were excluded because of ischemic heart disease (*n* = 2), body mass index (BMI) >35 kg/m2 (*n* = 4), and untreated depression (*n* = 1). Consequently, the final study cohort consisted of 30 subjects. The median age of the study participants (90% males) was 39.0 years (Table 1). Overall, 15 (50%) subjects had mild OSA (apnea-hypopnea index [AHI] <15 events per hour) and another 15 (50%) had moderate-to-severe OSA (AHI ≥15 events per hour). The median CCA-IMT in the entire study cohort was 0.76 mm, and 6 (20%) patients had a high CCA-IMT. Thirteen (43%) patients were obese; 9 (30%) had a high waist circumference. Nine (30%) patients had the MetS. The median 10-year CVD risk score was 3%, and four (13%) patients had intermediate CVD risk (≥10% and <20%). None had a high CVD risk. The baseline values of blood biomarkers are summarized in Table 1.

### Snoring sound analysis of full-night sleep in the home environment

Initially, we attempted to analyze 30 full-night snoring sounds in the home environment. However, we excluded 15 (50%) recordings because of unexpected noisy backgrounds (*n* = 6, [20%]), poor signal quality (*n* = 5, [17%]), and inadequate sleep time (*n* = 4, [13%]). We could not re-record the full-night snoring sounds because most of the participants underwent OSA surgery shortly thereafter. Table 1 shows differences in baseline characteristics between the included and non-included patients. All variables of interest were not significantly different except for the diastolic blood pressure (BP) of the included participants, which was significantly lower than that of the non-included patients (*p* = 0.037).

**Figure 1:**
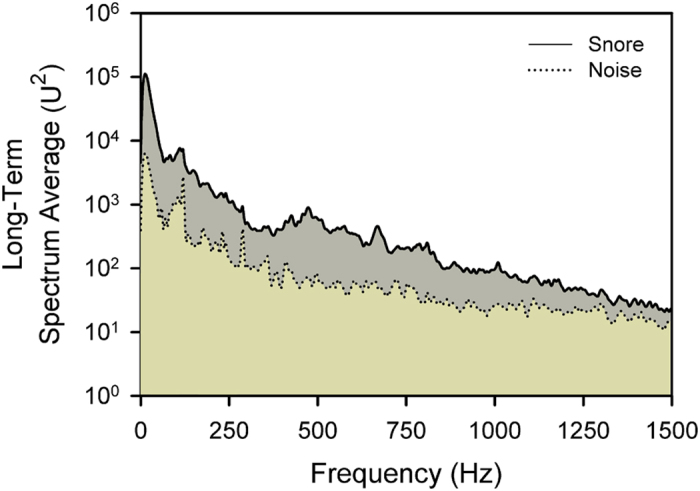
The long-term spectrum average of snores and noise. The net snore power (dark grey zone) was obtained by subtracting the long-term spectrum average of noise from that of snores for each frequency.

Figure 1 shows the pooled long-term spectrum average of snores (LTSA_snore) and of noise (LTSA_noise) of the 15 included participants. The snore spectrum revealed the strongest energy in the very-low-frequency range from 0 to 20 Hz, which is below the lowest frequency that humans can hear. Moreover, there were several identifiable energy peaks at approximately 100, 470, 670, 800, and 1000 Hz. The energy of noise was several to 20 dB lower than the snores for all frequencies, and the most prominent differences were revealed in the frequency range from 400 to 1000 Hz.

The median snoring index of 20 to 1500 Hz was 444 (348–768) events per hour, and the corresponding median energy was 60.7 (57.4–64.6) dB. There were no significant differences in snoring index and snoring sound energy in the gender (male *vs*. female), obesity (yes *vs*. no), MetS (yes *vs*. no), OSA (moderate-to-severe vs. mild), and CCA-IMT (high *vs*. low) groups (all *p* > 0.05).

### Correlations between CCA-IMT, BMI, MetS, CVD risk, OSA severity, and snoring

As shown in Table 2, CCA-IMT was not statistically significantly associated with BMI, MetS, CVD risk, AHI, minimal arterial oxygen saturation (SaO_2_), snoring index from 20 to 1500 Hz, snoring index from 20 to 652 Hz, and snoring sound energy from 20 to 652 Hz (*r* = 0.502 & 0.366, respectively; *p* = 0.056 & 0.242, respectively) before or after adjustment for age and sex in the included group (all *p* > 0.05). CCA-IMT was significantly correlated with mean SaO_2_ and snoring sound energy from 20 to 1500 Hz (*r* = 0.521; *p* = 0.046). However, the relationships between CCA-IMT, mean SaO_2_, snoring sound energy from 20 to 1500 Hz were not statistically significant after adjustment for age and sex (*r* = −0.423 & 0.401, respectively; *p* = 0.171 & 0.196, respectively).

### Post hoc analysis of snoring sound frequency with CCA-IMT

**Figure 2:**
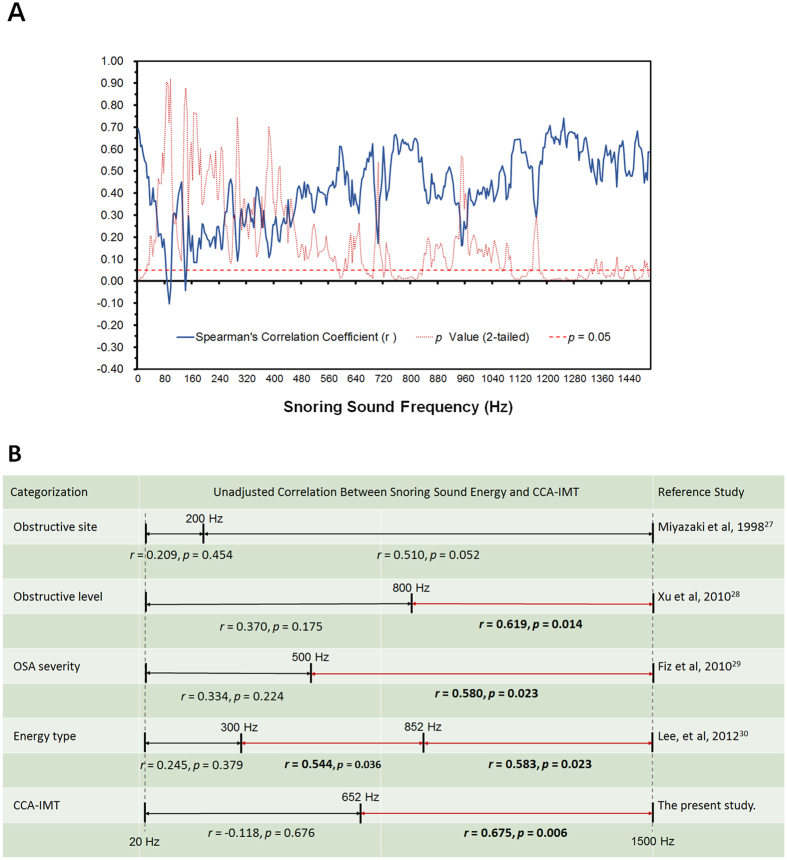
Correlations between snoring sound energy and common carotid artery intima-media thickness (CCA-IMT). (**A**) Spearman’s correlation coefficients and *p* values between snoring sound energy of each 4-Hz frequency band and CCA-IMT. (**B**) Associations of snoring sound energy of specific frequency bands with CCA-IMT. OSA, obstructive sleep apnea.

To further investigate associations of snoring sound energy in various frequency bands with CCA-IMT, we calculated the Spearman’s correlation coefficients for each 4-Hz frequency band (Fig. 2A). There were several clusters of frequency bands in which snoring sound energy significantly correlated with CCA-IMT, such as 584–612 Hz (*r* = 0.554, *p* = 0.032), 672–688 Hz (*r* = 0.579, *p* = 0.024), 744–832 Hz (*r* = 0.625, *p* = 0.013), and 1100–1500 Hz (*r* = 0.583, *p* = 0.023).

**Figure 3:**
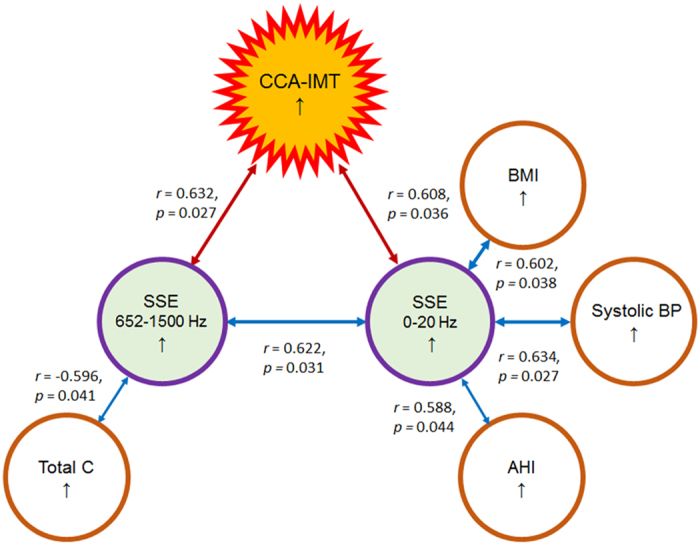
Spearman’s correlations of variables with common carotid artery intima-media thickness (CCA-IMT) after adjustment for age and sex in the included group. CCA-IMT is significantly related to snoring sound energy (SSE) of 652 to 1500 Hz and SSE of 0 to 20 Hz. Significant associations of variables of interest with other variables are also demonstrated. AHI, apnea-hypopnea index; BMI, body mass index; BP, blood pressure; C, cholesterol; LDL, low-density lipoprotein.

We also categorized snoring sound frequency into several smaller bands according to previous studies (Fig. 3B)^27^,^28^,^29^,^30^. Soft palate obstruction (≤200 Hz)/non-soft palate obstruction (>200 Hz)^27^, upper-level obstruction (≤800 Hz)/lower-level obstruction (>800 Hz)^28^, moderate-to-severe OSA (≤500 Hz)/simple snorer-to-mild OSA (>500 Hz)^29^, and low-frequency energy (≤300 Hz)/mid- frequency energy (300 to 852 Hz)/high-frequency energy (>852 Hz)^30^ were used as cut-off points. In general, the relationship between a snoring sound energy with a frequency >300 Hz and CCA-IMT was more significant than that between a snoring sound energy of 20 to 1500 Hz and CCA-IMT. Of note, a snoring sound energy of 652 to 1500 Hz was the most strongly positive energy factor correlated with CCA-IMT before (*r* = 0.675, *p* = 0.006) and after adjustment for age and sex (*r* = 0.632, *p* = 0.027; Table 2). Moreover, a snoring sound energy of 652 to 1500 Hz was also significantly inversely associated with TC (*r* = −0.596, *p* = 0.041) after adjustment for age and sex (Fig. 3). However, the corresponding snoring indexes were not related to CCA-IMT (All *p* > 0.05; data not shown).

Moreover, a snoring sound energy from 0 to 20 Hz also revealed a moderate-to-strong, positive, and significant correlation with CCA-IMT (*r* = 0.632, *p* = 0.011), systolic BP (*r* = 0.524, *p* = 0.045), mean SaO_2_ (*r* = −0.728, *p* = 0.003), and snoring sound energy from 652 to 1500 Hz (*r* = 0.632, *p* = 0.011). However, the associations of snoring sound energy from 0 to 20 Hz with CCA-IMT (*r* = 0.608, *p* = 0.036; Table 2), BMI, systolic BP, AHI, and snoring sound energy from 652 to 1500 Hz were significant after adjustment (Fig. 3), but the relationship with mean SaO_2_ (*r* = −0.423, *p* = 0.171) was not statistically significant after adjustment.

## Discussion

This study is the first to use acoustic analyses of full-night snoring sounds to investigate the associations between objective snoring sound frequency and energy and CCA-IMT in OSA patients. The snoring sound energy of specific frequency bands was significantly correlated with CCA-IMT; our findings further supported a positive association between subjective snoring severity and CCA-IMT. The present findings suggested that snoring may be a major determining factor of elevated CCA-IMT in OSA patients. Both sonic snoring sound energy from 652 to 1500 Hz and subsonic snoring sound energy from 0 to 20 Hz may be unique signatures of atherosclerosis in terms of elevated CCA-IMT.

The National Heart, Lung, and Blood Institute (NHLBI) of the National Institutes of Health in USA documented that older age (men, ≥45 y; women, ≥55 y) and overweight/obesity (BMI ≥ 25 kg/m^2^) were two major risk factors for atherosclerosis (http://www.nhlbi.nih.gov/health/health-topics/topics/atherosclerosis/atrisk). In this study, 40% (12/30) of them had older age and 57% (17/30) had overweight or obesity. Expectedly, our study subjects might not be at high risk for atherosclerosis. Moreover, despite participants with a low-to-intermediate 10-year CVD risk having no clinical cardiovascular events, their CCA-IMT was significantly higher than that of a large general population in Taiwan (0.76 mm vs. 0.68 mm, p* *<* *0.001; Wilcoxon signed-rank test)^31^. One of five study subjects had a high CCA-IMT (>1.04 mm) at a >2-fold risk of coronary events compared with CCA-IMT (<0.65 mm)^32^. Although the difference in 10-year CVD risk between low and high CCA-IMT was significant (2.0% [1.0–5.0%] *vs*. 7.0% [4.8% *vs*. 10.3%], *p* = 0.013; Mann-Whitney U test), their association was not statistically significant after adjustment for age and sex. Notably, a substantial number of middle-aged individuals with low CVD risk had extensive vascular atherosclerosis^33^. Therefore, carotid ultrasonography may help to reclassify CVD risk in OSA patients with low-to-intermediate risk^34^. Furthermore, CCA-IMT was not associated with OSA severity in this study. These findings are consistent with the results of a large longitudinal cohort study in which baseline AHI was a stronger predictor of future carotid plaques than CCA-IMT^35^.

Serum hs-CRP levels have been linked with increased AHI in OSA patients^36^,^37^,^38^. Intermittent hypoxia can induce adipocytes to secrete cytokines and activate atherosclerotic processes by increasing hs-CRP concentrations^39^. One recent study revealed a positive relationship between hs-CRP and CCA-IMT in OSA patients^37^. OSA patients have higher plasma levels of Hcy compared with control subjects^12^,^39^. Elevated Hcy concentrations are related to endothelial cell damage and impaired endothelial function and as well as to increased CCA-IMT in hypertensive patients^40^. Lee, *et al*.^16^ found that snoring was a significant predictor of carotid, but not femoral, atherosclerosis, and supposed that direct acoustic damage to the endothelium or the local induction of a proinflammatory response may result in the evolution of carotid atherosclerosis^22^,^23^,^24^. However, we found no significant relationship between these circulatory markers with snoring. This apparent discrepancy may be explained by the modest effect sizes of such risk factors in the pathogenesis of carotid atherosclerosis, which can yield negative findings in relatively small study cohorts.

**Figure 4:**
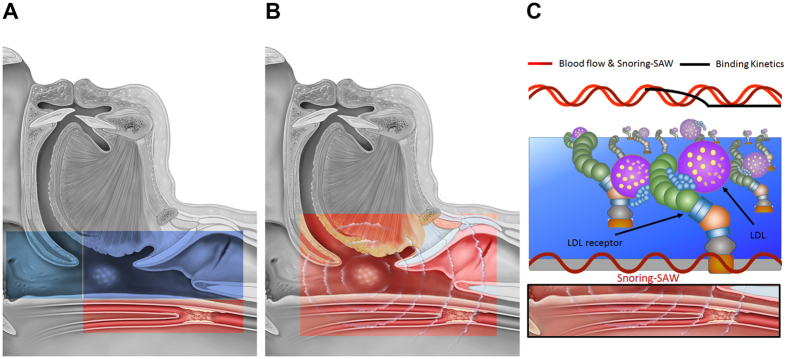
Possible explanations of snoring and increased common carotid artery intima-media thickness. (**A**) Lower-level obstruction induces local inflammation and oxidative stress. (**B**) Acoustic or vibratory energy of snoring generators causes nearby endothelial damage. (**C**) Surface acoustic wave (SAW) enhances binding kinetics and increases receptor-mediated endocytosis of low-density lipoprotein (LDL).

Objective snoring sound analysis indicated that snoring sound energies of specific frequency bands were associated with CCA-IMT. Accordingly, we explain these interesting findings in Fig. 4.

First, we try to classify the specific frequency bands according to the obstructive site^27^, obstructive level^28^, OSA severity^29^, and energy type^30^ (Fig. 2B). CCA-IMT was moderately-to-strongly correlated with snoring sound energies of ≥300 Hz and might be attributable to lower level obstructions (below the free margin of the soft palate), i.e., obstructed sites including the palatine tonsils, tongue base, lateral pharyngeal wall, larynx, individually or in combination, were associated with increased CCA-IMT. Because systemic inflammation/oxidative stress markers did not seem to be related to CCA-IMT in our subjects, this finding indicated the lower level obstructions might induce local inflammation and oxidative stress leading to the development of carotid artery thickening (Fig. 4A).

Second, our results implied that the specific snoring generators corresponding to snoring sound energy might promote early atherosclerotic processes in the CCA. For example, the fundamental frequency of palatal snoring was approximately 100 Hz^41^,^42^. The fundamental snoring sound frequencies of the tonsil, tongue base, and larynx were approximately 330 Hz, 1000 Hz, and 652 Hz, respectively^41^. Based on animal models, acoustic and/or vibratory energy were responsible for endothelial damage, endothelial dysfunction, and CCA athersclerosis^22^,^23^,^24^. In our study, we found significant relationships only within several specific frequency ranges. Moreover, CCA-IMT was strongly related to snoring sound energy from 652 to 1500 Hz, which might account for snoring caused by the tongue base and larynx. These findings suggested that snoring generators anatomically close to the carotid bifurcation may result in direct mechanical injury (acoustic trauma) to the endothelial cells of the CCA and adversely affect the CCA-IMT directly (Fig. 4B).

Finally, an inverse association between snoring sound energy from 652 to 1500 Hz and TC suggested that snoring may augment the receptor-mediated endocytosis of cholesteryl ester, resulting in decreased serum TC levels and increased CCA-IMT. Recently, a surface acoustic wave (SAW) has been applied to enhance binding kinetics for the highly sensitive detection of small molecules and cells^43^,^44^. The acoustic energy of a SAW can generate a micro-fluidic actuation and a variety of processes to reinforce the binding between receptors and ligands. Similarly, the local transmission of acoustic energy of snoring might also affect molecular binding to facilitate the deposition of cholesterol and atherosclerosis in the necks of snorers (Fig. 4C).

**Figure 5:**
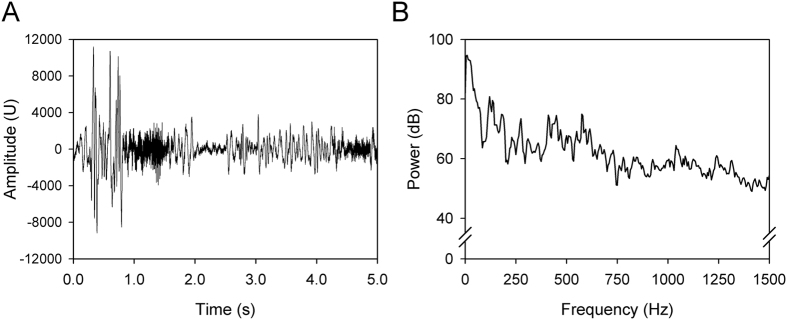
Presentation of sound signals. (**A**) The 5-sec acoustic signals of two typical snores. (**B**) The power spectrum analysis of the first snore.

In the present study, we found that snoring sound energy from 0 to 20 Hz was the strongest and was significantly associated with CCA-IMT. This very low, subsonic frequency snoring may involve acoustic oscillations resulting from obstructed airflow, and the amplitude of this snore energy was usually very large (≥10 times that of other frequency snoring sounds; Fig. 5). This energy could reflect the aerodynamics of a snoring event as revealed by its clinical importance of moderate-to-strong correlations with AHI, BMI, systolic BP, and CCA-IMT. Because the NHLBI documented that sleep apnea, overweight/obesity, and high BP were risk factors for CCA-IMT, we therefore suggested that snoring sound energy from 0 to 20 Hz may be a mediator of the association between these risk factors and CCA-IMT. Therefore, sound snoring energy in this frequency band, although inaudible to human ears, should not be overlooked and needs further research to confirm its role in contributing to atherosclerosis of the CCA.

The small sample size may limit the ability to detect a significant association between CCA-IMT, obesity, and MetS but was sufficient to detect associations between CCA-IMT and snoring sounds. Furthermore, the cross-sectional study design cannot demonstrate a direct causal link between snoring sound energy and increased CCA-IMT. A large-scale, longitudinal study is needed to clarify the exact atherosclerotic effects of snoring sound energy. Moreover, we did not investigate associations between other snoring sound variables such as snoring sounds variability (a measurement of continuity)^45^, and inter event silence (a measurement of long silent apnea)^46^ and CCA-IMT in the present study. Finally, this study was implemented in Chinese patients without generalization in ethnicity, which is thought to be an important predictor of CCA-IMT^47^. Therefore, our results needed to be externally validated for future generalization.

In conclusion, our results reveal significant correlations between snoring sound energy and increased CCA-IMT. Snoring sound energy from 652 to 1500 Hz is compatible with obstructive level and snoring generators in the respiratory tract close to the carotid artery bifurcation. Although more investigations are required to confirm the real mechanisms of CCA atherosclerosis and OSA, the acoustic energy of snoring in specific frequency bands may be one mechanism involved in the early process of CCA atherosclerosis.

## Methods

### Ethics statement

We conducted a prospective case series focusing on patients with OSA. Ethics approval was granted by the Institutional Review Board of the Linkou-Chang Gung Memorial Hospital (CGMH), Taoyuan, Taiwan (101-4981A3). All procedures were in compliance with the current regulations. All participants provided written informed consent.

### Study population

Patients with subjective symptoms of snoring and daytime fatigue who agreed to attend the study were prospectively recruited from the Department of Otorhinolaryngology Head and Neck Surgery at the CGMH between July 1, 2013 and June 30, 2014. Adult patients aged 30 to 60 years with newly diagnosed OSA, defined as an AHI ≥ 5 obstructive events per hour of sleep, were eligible for inclusion. Patients with (1) a BMI ≥35 kg/m^2^, (2) a history of CVD, stroke, or pulmonary disorder, (3) anxiety, depression, or psychiatric disorders, or (4) a prior history of tonsillectomy or OSA surgery were excluded.

### Study protocol

The demographic data, BMI, clinical symptoms, BP, and physical examinations of all patients were performed as described elsewhere^36^. Obesity was defined as a BMI ≥ 27 kg/m^2^ in line with the definition of the Taiwanese Health Promotion Administration, Ministry of Health and Welfare (http://health99.hpa.gov.tw/OnlinkHealth/Onlink_BMI.aspx). Daytime sleepiness was assessed using the Mandarin Chinese version of the ESS^48^. All of the participants underwent nocturnal polysomnography in our Sleep Center. Blood sample collection, carotid ultrasonography, and full-night snoring sound detection were performed within one month of polysomnography in all participants.

### Polysomnography

A standard in-lab polysomnography (Nicolet UltraSom System, Madison, WI, USA) was performed to document sleep parameters. Obstructive apnea was defined as the cessation of airflow for >10 sec with persistent respiratory effort, as seen in the ribcage or abdomen signals; hypopnea was defined as a ≥30% decrease in nasal pressure signal excursions for at least 10 sec accompanied by a desaturation of 4% or more from the pre-event baseline or as an arousal from sleep^49^. The AHI quantified the number of events per hour of scored sleep. Additionally, both mean and minimal SaO_2_ were recorded.

### Measurement of blood biomarkers for CVD

Patients with active infections, systemic inflammatory processes, or trauma were not tested until their conditions had diminished. After a 12-h fasting period, peripheral venous blood samples were obtained in the morning (between 7:00 and 7:30 a.m.). Whole-blood samples were collected in anticoagulant-containing tubes, centrifuged immediately, and then stored at 4 °C. Serum levels of TC, high-density lipoprotein cholesterol (HDL-C), LDL-C, TG, and hs-CRP were measured. Plasma levels of fasting plasma glucose (FPG) and Hcy were determined. All samples were processed by technicians who were blinded to the identity of samples.

Serum hs-CRP – an inflammatory marker – levels were determined by automated latex immunoturbidimetric assay (Nanopia CRP; Daiichi Pure Chemicals Co Ltd, Ryugasaki City, Japan) as described previously^35^. A serum level of hs-CRP ≥ 3 mg/L was considered to be increased.

Plasma Hcy – a marker of oxidative stress – levels were measured immediately after centrifugation using an automated colorimetric assay on a Hitachi 7600 modular chemistry analyzer (Hitachi Co Ltd, Tokyo, Japan) with the corresponding reagents (Sekisui Inc., Osaka, Japan)^50^. A serum level of Hcy≥12 μmol/L was considered to be increased. TC, HDL-C, LDL-C, and FPG were quantified using enzymatic assays. Levels of TG were assessed using a colorimetric assay. All biochemical markers were also measured on the Hitachi 7600 Modular Chemistry Analyzer (Hitachi, Tokyo, Japan).

### Diagnostic criteria for the MetS

According to the Adult Treatment Panel III of the National Cholesterol Education Program modified for Asians^5^, patients were diagnosed with MetS when they displayed three or more of the following five factors : 1) waist circumference ≥ 90 cm for Asian males or ≥ 80 cm for females (high waist circumference); 2) TG ≥ 150 mg/dL or the patient was on a specific drug treatment; 3) HDL-C < 40 mg/dL for males or the patient was on a specific drug treatment; 4) arterial blood pressure ≥ 130 or  ≥ 85 mmHg for systolic and diastolic blood pressure, respectively, or the patient was on antihypertensive drug treatment; 5) FPG ≥100 mg/dL or the patient was on a specific drug treatment.

### Calculation of 10-year CVD risk

The 10-year CVD risk was calculated using data from the Framingham Heart Study and was designed for adults aged 20 years or older who did not have heart disease or diabetes^51^. Baseline information, including age, sex, serum levels of TC and HDL-C, smoking, systolic blood pressure, and current usage of any medication for high blood pressure, were used to predict CVD risk expressed as percentiles over the next 10 years. An online calculator of CVD risk (http://cvdrisk.nhlbi.nih.gov/calculator.asp) provided by the National Heart, Lung, and Blood Institute at the National Institutes of Health (USA) was applied. The following categories were considered: low risk (<10%), intermediate risk (≥10% and <20%), and high risk (≥20%).

### Measurement of CCA-IMT

Carotid ultrasonography was performed by the same cardiologist (CYW) without knowledge of the subject’s clinical information to minimize observer bias. Images were electronically captured using a B-mode ultrasound system (Philips HDI 5000 System, ATL-Philips, Bothell, USA), and far-wall CCA-IMT was measured in the distal portion of each CCA in the proximal 1 to 2 cm of the carotid bulb in plaque-free areas^43^. The CCA-IMT measurement was repeated 6 times, and all results were averaged. The mean CCA-IMT of the right and left CCA-IMT values was used for statistical analysis, and ‘high CCA-IMT’ was considered to be ≥1.041 mm^32^.

### Detection and analysis of snoring sounds

For all participants, full-night snoring sounds were recorded using a portable digital sound recorder using linear pulse-code modulation (PCM-D50, Sony Electronics Inc., Tokyo, Japan) with two built-in high-performance electret condenser unidirectional dynamic microphones. These microphones were moved to a 90-degree X-Y pattern. We set the low cut filter switch to “OFF” in order to obtain snoring and breath sounds and the record level to “5”. This recorder was positioned 100 cm above the participant’s head as in our previous study^29^. Since distortion seldom occurred in full-night snoring sound recordings, we set the limiter switches to “OFF” to obtain original sound signals. One-kilohertz pure tones produced by a speaker at an intensity of 109.5 dB sound pressure level (SPL) in the sound-treated room were used for intensity calibration of the sound recorder. The subjects were asked to use a sound recorder and to sleep alone in a quiet bedroom (usually <40 dB SPL) without using hypnotics or sedatives. The standard Taiwanese bedroom sizes were 15−20 m^2^ in area and 2.4−3.2 m in height. The patients were asked to move their bed to maintain a minimum distance (>1/4 loading space) between the headboard and attached wall as previously described elsewhere^52^. Snoring sounds were recorded at a sampling rate of 44,100 Hz with a 16 bit A/D converter and were continued for at least 6 h for each participant. All data were stored in a computer and processed by digital recording software (LabVIEW, National Instruments Corp., Austin, TX, USA). As mentioned above.

To acquire the energy spectrum of snores, the sound signals were first reviewed using a playback system to identify the first snore. We manually removed all of the high frequency sounds (>3000 Hz) from acoustic analysis to reduce wall and/or ceiling reflections (ceiling effect)^52^. The 1-min signals before the first snore were considered background noise, and the root mean square (RMS) was obtained as a baseline to distinguish snores from noise. Figure 1 depicts the 5-sec acoustic signals of two typical snores (Fig. 5A). These snores showed a very low frequency (<20 Hz) but had large-amplitude vibrations in time periods from 0 to 1 sec and 2.5 to 4.5 sec. However, these snores also presented high-frequency sounds with small-amplitude vibrations in time periods from 1.0 to 1.5 sec and 4.5 to 5.0 sec. Power spectrum analysis of the first snore revealed the strongest energy at a frequency of 10 Hz. Several energy peaks occurred at higher frequencies of approximately 120 Hz, 450 Hz and 570 Hz (Fig. 5B).

Snoring sound analysis was performed throughout each recording using a 0.25-sec time window with no overlapping data. Windows whose signals had an RMS more than double the baseline, i.e., 6 dB or stronger than background noise, were considered snore epochs; all others were considered noise epochs. Consecutive snore epochs lasting 0.5 to 3 sec were considered snores, and the snore index was defined as the snore count per minute of analysis time. The power spectrum was acquired for each window using fast Fourier transformation (0 to 1500 Hz) with a frequency resolution of 4.0 Hz. In order to improve snore prediction, the RMS of noise was adaptively determined at 1-min intervals. All power spectra were divided into one of two groups: snore and noise. Additionally, the spectra in each group were subsequently averaged to obtain the LTSA_snore and LTSA_noise. Moreover, the net snore power (LTSA_net_snore), i.e., the power difference between snores and noise, was obtained by subtracting LTSA_noise from LTSA_snore for each frequency. The energy levels of various frequency domains (in dB) were acquired using a specially developed software program. To reduce the possibility of observer bias, snoring sound analysis was performed by a single author (GSL) who was blinded to the clinical data.

### Statistical analysis

Continuous and ordinal data are presented as medians and interquartile ranges and were compared using either the Wilcoxon signed-rank test (for comparing medians to hypothesized values) or the Mann-Whitney *U* test (for comparing two independent samples). Categorical data are presented as numbers and percentages and were compared using the Fisher’s exact test. We used Spearman’s correlation analysis to examine unadjusted associations between study variables. Adjusted partial correlations of significant variables and CCA-IMT for age and/or sex, the two most common risk factors^53^,^54^, were calculated. Two-tailed p values* < *0.05 were considered statistically significant. All statistical analyses were performed using IBM SPSS software (version 23.0; International Business Machines Corp., Armonk, NY, USA).

## Additional Information

**How to cite this article**: Lee, G.-S. *et al*. The Frequency and Energy of Snoring Sounds Are Associated with Common Carotid Artery Intima-Media Thickness in Obstructive Sleep Apnea Patients. *Sci. Rep*. **6**, 30559; doi: 10.1038/srep30559 (2016).

## Figures and Tables

**Table 1:** Patient baseline characteristics included and non-included in snoring sound analysis.

Variable	Overall (n = 30)	Included (n = 15)	Non-Included (n = 15)	*p* Value^A^
Age, y	39.0 (34.0–48.0)	37.5 (33.3–46.5)	43.0 (35.0–48.5)	0.567
Male sex, n (%)	27 (90)	12 (80)	15 (100)	0.224
BMI, kg/m^2^	25.9 (22.7–30.8)	25.5 (23.1–28.0)	26.0 (21.9–31.6)	0.744
Obesity, n (%)	13 (43)	6 (40)	7 (47)	1.000
WC, cm	83.8 (77.2–91.4)	82.5 (77.0–83.8)	85.1 (78.8–94.5)	0.250
High WC, n (%)	9 (30)	3 (20)	6 (40)	0.427
Smoking, n (%)	10 (33)	4 (27)	6 (40)	0.700
ESS	9.0 (6.0–11.0)	7.5 (3.8–11.0)	9.0 (7.0–11.0)	0.227
AHI, events/h	18.3 (7.4–39.1)	16.5 (9.9–36.5)	19.1 (6.8–77.6)	0.744
M-S OSA, n (%)	15 (50%)	7 (47)	8 (53)	1.000
Mean SaO_2_, %	95.0 (90.0–96.0)	95.0 (94.0–96.0)	93.7 (89.5–95.5)	0.125
Minimal SaO_2_, %	84.0 (72.0–87.0)	84.0 (77.3–88.0)	85.0 (65.5–86.5)	0.839
Systolic BP, mmHg	136.0 (119.0–155.0)	129.5 (116.5–146.8)	142.0 (118.0–155.5)	0.389
Diastolic BP, mmHg	79.0 (69.0–95.0)	73.0 (64.8–90.0)	92.0 (74.0–96.0)	0.037
Total cholesterol, mg/dL	188.0 (166.0–241.0)	192.0 (166.0–233.3)	188.0 (171.0–241.5)	0.902
Triglyceride, mg/dL	123.0 (93.0–224.0)	120.5 (102.8–179.8)	150.0 (60.5–289.0)	0.715
HDL-C, mg/dL	45.0 (38.0–49.0)	47.0 (40.8–50.8)	42.0 (35.5–47.5)	0.217
LDL-C, mg/dL	116.0 (102.0–146.0)	126.5 (102.0–153.0)	116.0 (94.0–143.0)	0.683
FPG, mg/dL	90.0 (82.0–95.0)	93.0 (84.5–105.3)	87.0 (78.0–91.5)	0.081
MetS, n (%)	9 (30)	2 (13)	7 (47)	0.109
10-year CVD risk, %	3.0 (1.0–5.0)	2.5 (1.0–5.0)	5.0 (1.0–9.5)	0.202
Intermediate CVD risk, n (%)	4 (13)	0 (0)	4 (27)	0.100
hs-CRP, mg/L	1.76 (0.74–2.99)	1.76 (1.36–3.51)	2.00 (0.51–2.47)	0.591
High hs-CRP, n (%)	7 (23)	4 (27)	3 (20)	1.000
Hcy, μmol/L	9.10 (8.50–10.70)	9.05 (8.38–9.85)	9.69 (8.25–10.90)	0.425
High Hcy, n (%)	10 (33)	3 (20)	7 (47)	0.245
CCA-IMT, mm	0.757 (0.667–0.877)	0.754 (0.660–0.827)	0.815 (0.674–1.210)	0.389
High CCA-IMT, n (%)	6 (20)	2 (13)	4 (27)	0.651

Note: Data are median (quartile 1 to 3) or n (%) as appropriate.

^A^Mann-Whitney U and Fisher’s exact tests.

AHI, apnea-hypopnea index; BMI, body mass index; BP, blood pressure; CCA-IMT, common carotid artery intima-media thickness; CVD, cardiovascular disease; ESS, Epworth Sleepiness Scale; FPG, fasting plasma glucose; Hcy, homocysteine; HDL-C, high-density lipoprotein cholesterol; hs-CRP, high-sensitivity C-reactive protein; LDL-C, low-density lipoprotein cholesterol; MetS, metabolic syndrome; M-S OSA, moderate-to-severe obstructive sleep apnea; SaO_2_, arterial oxygen saturation; WC, waist circumference.

**Table 2:** Correlations with or without adjustment for age and sex of common carotid artery intima-media thickness in patients with obstructive sleep apnea in the included group.

Variable	Unadjusted^A^		Adjusted^B^	
	*r*	*p* Value	*r*	*p* Value
Age	0.441	0.100	0.527	0.053
Male sex	0.116	0.681	0.259	0.372
Body mass index	0.443	0.098	0.350	0.264
Waist circumference	−0.155	0.581	−0.034	0.916
Epworth Sleepiness Scale	0.135	0.645	0.180	0.575
Smoking	−0.314	0.254	−0.310	0.327
Apnea-hypopnea index	0.143	0.612	0.170	0.598
Mean arterial oxygen saturation	−0.712	0.004	−0.423	0.171
Minimal arterial oxygen saturation	0.121	0.680	0.170	0.598
Systolic blood pressure	0.073	0.795	0.357	0.254
Diastolic blood pressure	0.007	0.980	0.211	0.511
Total cholesterol	−0.232	0.401	−0.247	0.438
Triglyceride	−0.424	0.131	−0.499	0.099
High-density lipoprotein cholesterol	−0.182	0.517	−0.374	0.231
Low-density lipoprotein cholesterol	−0.255	0.379	−0.076	0.813
Fasting plasma glucose	−0.077	0.785	−0.307	0.331
Metabolic syndrome	−0.091	0.748	−0.174	0.588
10-year cardiovascular disease risk	0.035	0.901	−0.255	0.424
High-sensitivity C-reactive protein	−0.387	0.171	−0.462	0.130
Homocysteine	0.397	0.302	0.022	0.945
Snoring index of 20 to 1500 Hz	−0.089	0.752	−0.019	0.953
Snoring sound energy of 20 to 1500 Hz	0.521	0.046	0.401	0.196
Snoring index of 20 to 652 Hz	−0.089	0.752	−0.019	0.953
Snoring sound energy of 20 to 652 Hz	0.502	0.056	0.366	0.242
Snoring index of 652 to 1500 Hz	−0.089	0.752	−0.019	0.953
Snoring sound energy of 652 to 1500 Hz	0.675	0.006	0.632	0.027
Snoring sound energy of 0 to 20 Hz	−0.089	0.752	−0.019	0.953
Snoring sound energy of 0 to 20 Hz	0.632	0.011	0.608	0.036

^A^Spearman correlation test.

^B^Partial correlation test.
